# ‘Sawa Aqwa’ (Stronger Together): A multi‐site randomized controlled trial of a brief family systemic intervention for adolescent mental health in Lebanon

**DOI:** 10.1111/jcpp.70174

**Published:** 2026-06-04

**Authors:** Tania Bosqui, Felicity L. Brown, Sally Farah, Joseph Elias, Maliki E. Ghossainy, Anas Mayya, Diana Abo Nakkoul, Bryony Walsh, Sarah Chreif, Ahmad Einein, Bassel Meksassi, Roula Abi Saad, Hady Naal, Michael Donnelly, Theresa S. Betancourt, Alan Carr, Eve S. Puffer, Estefania Hanna, Hasan Mansour, Farah Semaan, Rabih El Chammay, Mark J.D. Jordans

**Affiliations:** ^1^ Department of Psychology American University of Beirut Beirut Lebanon; ^2^ Trinity Centre for Global Health, Trinity College Dublin Dublin Republic of Ireland; ^3^ Research and Development Department War Child Alliance Amsterdam The Netherlands; ^4^ Amsterdam Institute of Social Science Research, University of Amsterdam Amsterdam The Netherlands; ^5^ War Child Alliance Beirut Lebanon; ^6^ Wheelock College of Education and Human Development, Boston University Boston MA USA; ^7^ Terre des Hommes Italy Beirut Lebanon; ^8^ Danish Refugee Council Beirut Lebanon; ^9^ Child Protection United Nations Children's Fund (UNICEF) Beirut Lebanon; ^10^ Global Health Institute, American University of Beirut Beirut Lebanon; ^11^ Centre for Public Health, School of Medicine, Queen's University Belfast Belfast UK; ^12^ Boston College School of Social Work Chestnut Hill MA USA; ^13^ School of Psychology, University College Dublin Belfield, Dublin Republic of Ireland; ^14^ Department of Psychology & Neuroscience Duke Global Health Institute, Duke University Durham North Carolina USA; ^15^ Ministry of Public Health Beirut Lebanon

**Keywords:** Humanitarian, mental health and psychosocial support, family, adolescents, randomized controlled trial, low‐ and middle‐income (LMIC) countries, Lebanon, refugees, armed conflict

## Abstract

**Background:**

There are no evaluated family‐based mental health and psychosocial support (MHPSS) interventions for adolescents in Southwest Asia (known as the Middle East), and few whole‐family interventions in low‐ and middle‐income countries, despite consistent evidence for the impact of family support on mental health and well‐being. This study aims to evaluate the effectiveness of a brief family systemic mental health intervention, deliverable by non‐specialists in mental health.

**Methods:**

We conducted an assessor‐blind type I hybrid effectiveness‐implementation multi‐site randomized controlled trial comparing the locally developed family intervention to a waitlist control group for randomly allocated families residing in North Lebanon and Beqa’a governorates. Eligible families presented with medium‐to‐high risk for child protection concerns (abuse, neglect, child labor, early marriage) and had at least one adolescent aged 12–17 who demonstrated psychological distress. Outcomes at the family, caregiver, and adolescent level were measured pre‐ and post‐intervention, and at 3‐month follow‐up.

**Results:**

Intent‐to‐treat analyses found a significant between‐group effect of the intervention on adolescent‐reported family functioning, caregiver mental health, and parenting. No change was found for adolescent psychological distress. Further analyses found effects on adolescent well‐being for those who completed the intervention, and that father attendance was associated with better outcomes for adolescent well‐being in the intervention group. No other significant moderators were found. At the 3‐month follow‐up for the intervention condition, family functioning and caregiver well‐being significantly dropped from endline.

**Conclusions:**

The study demonstrates mixed results for a non‐specialist‐delivered family‐systemic intervention developed in the context of humanitarian crises in Lebanon. While the intervention did not result in benefits in adolescent‐reported symptoms of psychological distress, the intervention group did show greater improvements than the control group on a number of other outcomes, showing the potential impact of working with the wider family system to support adolescents in humanitarian settings.

## Background

Mental health and psychosocial support (MHPSS) interventions have a growing evidence base in humanitarian settings (Barbui et al., 2020), but major gaps remain in addressing systemic, collective and social determinants of mental health (Bosqui, 2020). International humanitarian guidance has increasingly called for a greater focus on family‐based systemic MHPSS interventions that address the wider social context of adolescents affected by humanitarian disasters (Elrha, 2020). Adolescence is a unique and critical period of development with a lasting impact on adult mental health and well‐being. In humanitarian settings, adolescents are strongly impacted by their wider social ecological environment (Fayyad et al., 2017). Within the social ecological model (Bronfenbrenner, 1979), the microsystem family influences of family violence, poor parent mental health, parent–child relationships and attachment, family functioning, and parenting style are strongly associated with adolescent mental health and wellbeing (McEwen et al., 2022). The family system, in turn, is commonly impacted by structural (macrosystem) determinants such as poverty, stigma, and discrimination (Farah et al., 2026). At the same time, families' access to quality specialist mental health care is constrained by limited service availability, fueled by a major gap in trained mental health professionals across humanitarian and low‐resource settings (Jordans & Kohrt, 2020). In addition, one of the major limitations of existing MHPSS delivery for adolescents and their caregivers is the lack of engagement with fathers (Bosqui et al., 2024). This is despite clear evidence that fathers' mental health, well‐being, and parenting practices are strongly linked to both adolescent and maternal mental health (Papaleontiou‐Louca & Al Omari, 2020), as well as to the family's attendance and engagement in services (Baran & Sawrikar, 2024). Families’ low help‐seeking behavior may also occur due to other barriers, including stigma (El Masri, Chaar, Elias, Meksassi, & Ali, 2024) and perception of limited relevance given the complex social and structural barriers facing families (Roberts et al., 2022).

Few family and parenting interventions in humanitarian settings have been evaluated for adolescent mental health and well‐being (Bosqui et al., 2024), including in the Southwest Asian region (known as the Middle East) where there are ongoing protracted conflicts and limited mental health resources. Family‐based interventions in other contexts (high‐income, non‐humanitarian settings) have demonstrated good evidence for effectively promoting adolescent mental health (Carr, 2019). There is a need for MHPSS interventions that are developed in‐context, can be delivered by the existing workforce, align with perceived needs of families, and address the wider social ecology of adolescents in humanitarian settings (Elrha, 2020). The IASC (2007) guidance for MHPSS in emergencies recommends implementing not just specialist interventions, but also family and community interventions that can strengthen the social ecology of affected children, and can be delivered by ‘non‐specialist’ or lay providers. This model provides a mechanism to improve the reach and scale of interventions despite limited trained specialists (Sangraula et al., 2025). In addition, research is needed on what works for whom, given the current gap in knowledge and the implications for practice and policy (Brake, Willems, Steen, & Dückers, 2022). Research in non‐humanitarian contexts has identified mixed evidence for the role of age, gender, level of trauma exposure, and socioeconomic status on treatment outcomes (Gergov et al., 2024), as well as evidence for the role of father engagement in improving the impact of caregiver‐focused interventions (Gonzalez et al., 2023).

**Figure 1:**
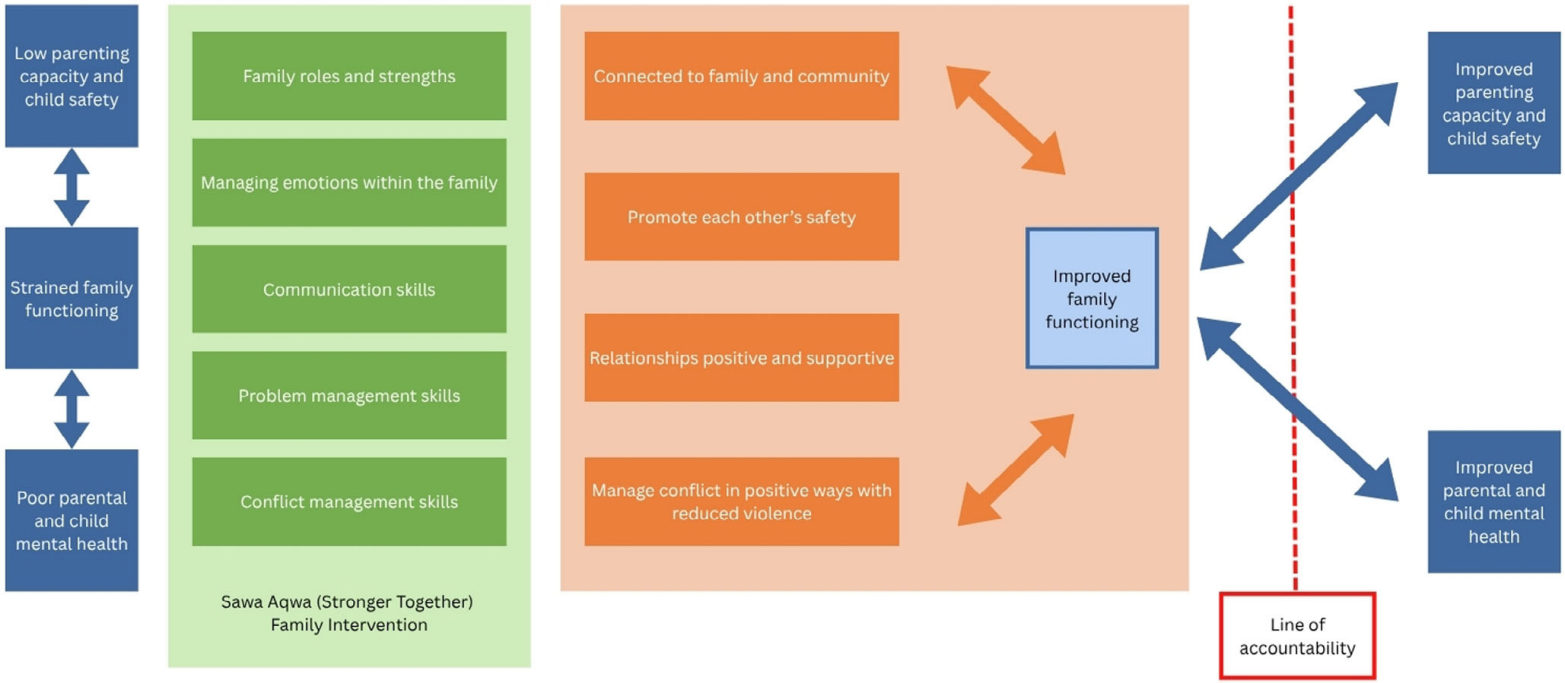
Theory of change for the Sawa Aqwa (Stronger Together) family intervention

Based on the existing evidence base, we developed a family systemic psychosocial support intervention. We drew on (a) an analysis of key intervention components and implementation models found in effective family interventions (Bosqui et al., 2024); (b) interviews with adolescents and their families in Lebanon (Farah et al., 2026); (c) a series of collaborative workshops with non‐governmental organizations, UNICEF, academic, and governmental partners, local and international experts; and (d) a Community Advisory Board (CAB) from the target communities. The intervention, named ‘Sawa Aqwa’ (meaning stronger together in Lebanese Arabic), is a six‐session whole family intervention. It was designed to be delivered by non‐specialists in mental health through the existing child protection system in Lebanon. The Theory of Change, displayed in Figure 1, draws on systems theory, attachment theory and the social ecological model. These included assumptions that adolescent and caregiver mental health are interlinked through attachment, emotional and relational patterns, that family systems can protect and promote individual mental health, and that family systems are influenced by macro‐level stressors and social injustices (further details can be found in Brown et al., in prep). Its primary aim (within the ‘line of accountability’ for the intervention) is therefore to improve family functioning, in order to promote adolescent and caregiver mental health and well‐being, parenting and safety. Change in family functioning is assumed to improve adolescent mental health and well‐being, caregiver mental health and well‐being and parenting. This is thought to function in the same way as the mechanisms identified in our formative qualitative work with families, where family functioning, adolescent, and caregiver mental health, and parenting, were closely intertwined (Farah et al., 2026). Promoting family functioning maps onto the objectives of the intervention that includes connecting to family, promoting each other's safety, building positive and supportive relationships, and managing conflict in positive ways with reduced violence.

### Aims and hypotheses

The primary aim of this study was to assess the effectiveness of the family intervention on adolescent‐reported symptoms of psychological distress. The secondary aim was to assess effectiveness on caregiver‐reported psychological distress and parenting, as well as adolescent‐ and caregiver‐reported family functioning and well‐being. Furthermore, we aimed to explore possible treatment moderators (e.g., age, gender, past traumatic exposures, level of adversity, income, and father attendance). Our hypotheses were that (1) adolescents and caregivers assigned to receive the family intervention would show significantly greater improvements in primary and secondary outcome measures compared to the waitlist group at post‐intervention; (2) that improvements will be maintained at the 3‐month follow up time point for the treatment condition; and that (3) age, gender, past traumatic exposures, level of adversity, income, and father attendance will moderate the primary outcome for the intervention group at post‐intervention.

## Methods

### Design

We conducted an assessor‐masked Type I hybrid effectiveness‐implementation multi‐site superiority randomized controlled trial (RCT) in Lebanon, comparing the newly developed family intervention (Sawa Aqwa—Stronger Together) to a waitlist control group. The superiority Type 1 design aimed to focus on testing the intervention effectiveness compared to a control condition, with a secondary focus on implementation science. Full details of the protocol have been published (Brown et al., 2022). The implementation component included identifying intervention acceptability, adoption, appropriateness, complexity, penetration, reach, and sustainability through session data, implementation logs, and qualitative interviews with families, facilitators, and supervisors, as well as feasibility, fidelity, and cost through individual family versus multi‐family format comparisons, session checklists and structured observations, and tracking implementation costs. Implementation outcomes will be reported separately (Bosqui et al., in prep). The trial was registered with the National Mental Health Program in Lebanon (registered 19 October 2021), the Lebanese Clinical Trial Registry (registered 13 October 2021, reference: LBCTR2021104870), and the ISRCTN (registered 1 November 2021, reference: ISRCTN13751677).

We assessed outcomes on family, adolescent, and caregiver measures at baseline (T0) and post‐intervention (T1), and at a 3‐month follow‐up for the treatment arm (T2). A waitlist comparison condition was chosen in order to enable us to deliver a group‐format version of the intervention with the control arm after T1 assessments were complete. The CONSORT checklist is provided in Table S1.

### Setting

The trial was conducted in Lebanon, a lower‐middle‐income Southwest Asian country suffering through the world's worst economic collapse. This has seen the local currency lose over 90% of its value in less than 5 years, and plunged over two thirds of the population into poverty (World Bank, 2024). Recent estimates also show that one in four people in Lebanon is a refugee or person in need of international protection (UNHCR, 2023). In addition, the population faced the Beirut port explosion in August 2020, and since October 2023, a cross‐border conflict with Israel that escalated into a full‐blown war in September 2024. A fragile ceasefire is in place at the time of writing, with ongoing daily bombardments by Israel (OHCHR, 2025). In this context, adolescents in Lebanon have worsening psychological distress, with probable anxiety disorders jumping from around 13% to 64% in the last few years (Maalouf et al., 2016, 2022). Despite this, less than 10% of adolescents in Lebanon with a probable mental disorder access mental health services (Maalouf et al., 2016). For this study, the intervention was implemented by three implementing partners (i.e., three sites) operating in two governorates of Lebanon‐ primarily in urban areas in North Lebanon (War Child [WC], Danish Refugee Council [DRC]) and in informal tented settlements in agricultural rural areas in north Beqa’a (Terre Des Hommes Italy [TDH]). Each implementing partner had existing child protection programming in these areas.

### Participants

Families were included if they: (i) had an adolescent aged 12–17 years (male and female); (ii) were identified as medium to high risk for child protection concerns by the implementing organization (defined for this study as being ‘vulnerable to a protection risk but not with imminent risk’ and includes risks such as abuse, neglect, child labor, early marriage); (iii) gave assent and parental/legal guardian consent to take part in trial procedures; and (iv) had at least one adolescent scored above the cut‐off on the Pediatric Symptom Checklist (PSC‐17, Murphy et al., 2016) for general psychological distress at screening (using the cut‐off identified by a validation study in the same population, Brown et al., 2024). Families of any nationality and background were eligible, and families of any composition (including single‐ or dual‐headed households) provided that an adult legal guardian was present.

Participants were excluded if they met any of the following criteria: (i) imminent child protection risk requiring immediate case management at the time of recruitment, or if they had severe psychiatric disturbance or risks requiring specialist mental health services (assessed by partners as part of usual routine assessment and referral systems); (ii) unaccompanied and separated minors, and adolescents who were married, due to challenges with the legal consent of guardians; and (iii) engaged in case management at the time of outreach, as those in case management were deemed likely to be receiving sufficient services. If the need for case management arose during the intervention, families were referred as usual and could remain in the intervention and study.

The sample size calculation was based on a two‐group comparison of the primary outcome at the post‐intervention time point. Assuming a 5% two‐tailed significance test and 80% power, it was estimated that data from 270 participants would need to be available at the post‐intervention timepoint to detect an effect size of Cohen's *d* = .25. Allowing for 30% attrition, this corresponded to an overall sample size of 351 required at enrolment.

**Figure 2:**
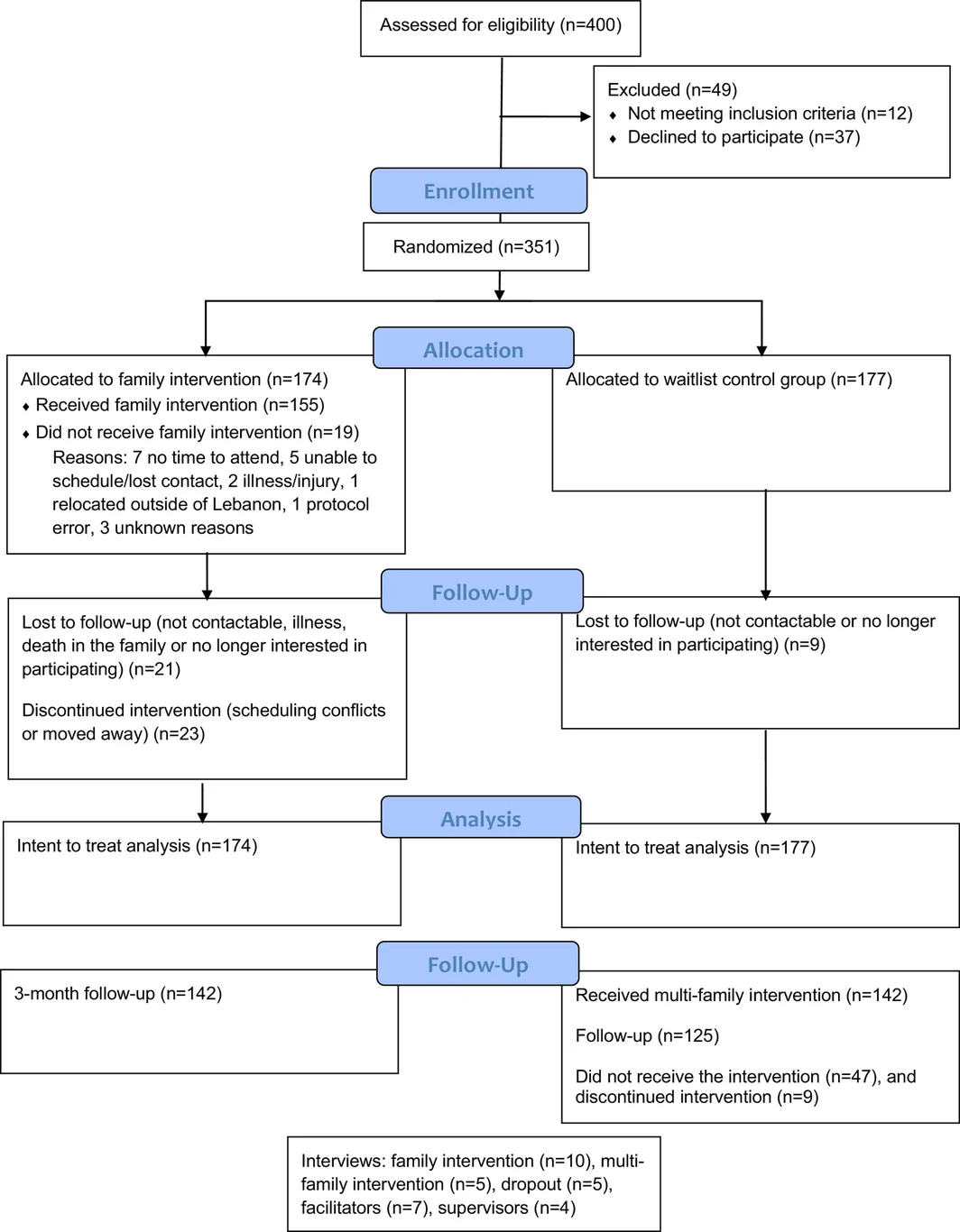
CONSORT flow diagram

Study enrollment began on 3 November 2021 until 8 April 2022. We screened 400 families to recruit the 351 families needed (87% screened in). There were 351 index adolescents and 388 caregivers assessed at baseline; 82.47% of caregivers were mothers. As shown in Figure 2, out of these families, 174 were randomized to the family intervention group and 177 families to the waitlist control group. At the 3‐month follow‐up, 142 families were assessed in the family intervention group, and another 142 from the control group received the multi‐family version of the intervention. This is a retention rate of 80.91%. Eighty‐nine percent of families in the intervention group attended at least one session. Over 70% of families completed the intervention (attended 4 or more sessions), while just over a third of fathers (39%) attended the intervention (1 session or more).

### Procedure

Recruitment was conducted by implementing partners through community outreach teams, guided by a standard script. After consent/assent, screening and assessment interviews were conducted by Research Assistants over the telephone using RedCap and KoBo software on tablets, with all adolescents and caregivers in the household. Fathers and mothers were both contacted to ensure each was invited. Where more than one adolescent screened positive, the adolescent scoring the highest was selected as the ‘index adolescent’, and only this adolescent completed outcome measures at T0 and the remaining timepoints, though all family members were invited to join the intervention.

Research assistants were undergraduate or graduate psychology students who received training and practice sessions including phone delivery, sensitive interviewing, managing distress and risk management, research ethics, gaining informed consent and assent, study procedures and record keeping, provision of compensation, risks of bias in collecting quantitative data, adverse events reporting procedures, and data management. Ongoing monitoring of assessors' competency and consistency was conducted through regular supervision by TB.

Assessments at T0 and T1 were conducted within 4 weeks of initiation/completion. Families received 25,000 LBP (approximately 7–10 USD at the time) per assessment to reimburse them for phone data usage and time.

### Randomization

Randomization to intervention or waitlist control group occurred after T0 assessments were completed, stratified by site. The unit of randomization was families, and they were randomly allocated to intervention or waitlist with a 1:1 ratio. The randomization sequence was computer‐generated by a statistician independent of the study using Research Randomizer (randomizer.org, Urbaniak & Plous, 2013), a computer‐based pseudo‐random number generator using the ‘Math.random’ method within the JavaScript programming. To ensure approximately equal randomization as enrolment progressed, blocking was used with block sizes unknown to the research team. The statistician allocated each eligible family ID to a study arm based on the sequence and shared the allocation with the local study team.

### Outcome measures

The majority of measures had been used in Lebanon previously (Brown et al., 2019; Pluess et al., 2024) except for two measures—the Systemic Clinical Outcome and Routine Evaluation (SCORE) Index of Family Functioning (Stratton et al., 2014) and the Difficulties in Emotion Regulation Scale‐Short Form (DERS‐SF, Bjureberg, Ljótsson, Tull, Hedman, & Sahlin, 2016). These were translated into non‐formal Arabic understood by participants in the region (i.e., Syrians, Lebanese, and Palestinians) following an adaptation of recommended procedures for cross‐cultural research (van Ommeren et al., 1999). Steps involved: forward translation to Arabic by four team members, independent back translation to English by two people, workshops with English‐speaking and bilingual team members to review the translations and ensure they retained the original English meaning, cognitive interviews with CAB members to assess comprehensibility, completeness, relevance, and acceptability, review workshops to adjust as needed, and pilot testing with target populations.

#### Primary outcome

The primary outcome was adolescent‐reported psychological distress, assessed by the Pediatric Symptom Checklist 35 (PSC‐35, Jellinek et al., 1999). The PSC‐35 consists of 35 symptoms (including internalizing, externalizing, somatic, social, and academic difficulties), in statements such as ‘worries a lot’, rated for their frequency on a 3‐point scale ranging from 0 (*never*) to 2 (*often*). The total PSC‐35 score is obtained by summing scores of individual items and ranges from 0 to 70, with higher scores indicating poorer mental health.

#### Secondary outcomes

##### Adolescent‐reported outcomes

Adolescent‐reported well‐being was measured with the WHO‐5 Wellbeing Index (WHO, 1998; Topp, Østergaard, Søndergaard, & Bech, 2015). The WHO definition of well‐being encompasses quality of life and a sense of meaning and purpose. The WHO‐5 consists of 5 statements, such as ‘my daily life has been filled with things that interest me’, asking participants to rate their answers over the last week on a scale from 0 (*none of the time*) to 5 (*all of the time*). Total scores range from 0 to 25, with higher scores indicating better well‐being.

Adolescent‐reported emotional regulation was measured with the DERS‐SF (Bjureberg et al., 2016). The DERS‐SF consists of 18 statements, such as ‘when I’m upset, I lose control over my behavior’, with participants rating their answers in the past week on a scale from 1 (*almost never*) to 5 (*almost always*). Total scores range from 18 to 90, with higher scores indicating more difficulty in emotion regulation.

Adolescent‐reported family functioning was measured with the SCORE (Stratton et al., 2014). The 15‐item measure asks participants to rate whether the item describes their family on a scale from 1 (*not at all*) to 5 (*very well*) in the past week, with items such as ‘when people in my family get angry they ignore each other on purpose’. Total scores range from 15 to 75, with a lower score indicating higher family functioning.

##### Caregiver‐reported outcomes

Caregiver psychological distress was measured using the Kessler Psychological Distress Scale (K6, Kessler et al., 2002). The K6 consists of six questions asking about mental health, such as whether the person has felt ‘nervous’ or hopeless' in the previous week, which are scored on a scale from 1 (*all of the time*) to 5 (*none of the time*). Total scores range from 6 to 30, calculated by summing individual items. Lower scores indicate higher levels of psychological distress.

Caregiver‐reported parenting skills were measured with the Brief Parenting Scale developed by War Child Alliance. The 26‐item scale developed in Lebanon as part of a parenting intervention trial (Miller et al., 2023) asks parents to rate their parenting practices (including items related to parental warmth, harsh discipline, and positive parenting) over the last week on a scale from 1 (*rarely*) to 3 (*often*). Total scores range from 26 to 78, with higher scores indicating healthier parenting practices.

Caregiver‐reported emotional regulation and family functioning were measured with the same measures as those of adolescents.

##### Moderator measures

Age, gender, working status, past traumatic exposures, level of adversity, and income were considered as potential moderators based on past studies on MHPSS in emergencies (Barbui et al., 2020). In addition, given our efforts to engage fathers in the intervention, we measured father attendance session‐by‐session to include as a moderator for the primary outcome in the intervention group. To measure exposure to potentially traumatic events and daily adversity in adolescents as a demographic characteristic, and possible moderator of treatment effects, we developed a 36‐item inventory to be asked to caregivers. The list was adapted from a checklist used in a recent RCT in a similar population (Brown et al., 2019), with existing items being adjusted and daily stressors being added based on formative research for this trial (Farah et al., 2026).

### Intervention

Sawa Aqwa is a six‐session family intervention delivered by non‐specialist facilitators once weekly for 2 hr (90 min with the whole family and 30 min with the caregivers). Families were defined as all caregivers within the household and children meeting the inclusion criteria. A booster session was provided after approximately 1 month. The intervention components cover these main skills: identifying family values, goals, challenges, and strengths; emotion regulation; communication; problem management; and managing disagreements. The caregiver component included psychoeducation and positive parenting for adolescents, and the booster session included a recap as well as mechanisms to increase social support (see Table S2 for more details). Key features of the intervention include that it: (i) involves the whole family; (ii) is transdiagnostic in nature, meaning that it targets families experiencing psychological distress broadly; (iii) is designed specifically for families living in communities characterized by adversity and acknowledges their lived reality; and (iv) can be safely delivered by non‐specialists who receive robust training and ongoing supportive supervision. It was delivered through the child protection system, a widely accessible pathway to focused MHPSS in the region, with potential for delivery at scale. The intervention is intended to be delivered face‐to‐face in a home visit or in a community center; however, due to safety concerns or restrictions related to COVID‐19, a remote telephone modality was also employed as needed.

### Facilitator selection, training and supervision

Intervention facilitators were all female non‐specialist providers. They were recruited through implementing partners using the same criteria, including (1) a university degree in psychology, social work, or a related field, and (2) at least 2 years' experience providing psychosocial support to both children and caregivers. Facilitators received 9 days of training in basic counseling skills and competencies. This included: competencies for working with adolescents, caregivers, and the family system; delivering the content of the intervention based on the manual; and self‐care. Trainings were informed by formative qualitative work to ground skills in the local context and culture. For example, including local idioms of distress like *daghet nafsi* (psychological pressure in Arabic), the acknowledgment and containment of massive adversity and injustice related to the socio‐economic and political context, and the use of coping strategies such as *saber* (active patience in Arabic). At the end of the training, all facilitators underwent a structured assessment of competencies in order to be eligible to implement the intervention. Weekly supervision was provided by a local masters‐level psychologist (JE), who received regular supervision with the principal investigators (FB, TB) and a locally licensed clinical psychologist to monitor quality and fidelity of implementation, give input on challenging cases, and provide support in supervision.

### Ethics and trial management

The trial received local ethical approval from the Institutional Review Board of the American University of Beirut (Protocol ID: SBS‐2021‐0102). A trial management committee consisting of principal investigators, co‐investigators, partner staff, and the research coordinator met regularly through a series of weekly, fortnightly, and monthly meetings to monitor the study. All adverse events (e.g., injuries on the way to sessions, increase in distress) and serious adverse events (e.g., suicide attempts, violence) were recorded by the research team and reported to a Data Safety Management Board (DSMB). DSMB meetings were facilitated monthly by TB, and the board consisted of 3 local doctoral‐level mental health professionals, external to the study. The local study coordinator was responsible for ensuring timely follow‐up of all reports. In total, 71 children were referred for case management during the study period (*n* = 38, 22.09% in the intervention group and *n* = 33, 19.19% in the control).

### Masking

Participants and implementation staff were not masked to group allocation, but assessors were masked to group allocation of families at T1 and T2, and operated independently from intervention facilitators. One of the co‐PIs (FB) and the statistician were also masked to the treatment condition. When the allocation was accidentally revealed (four times due to research team error and twice due to family members mentioning the intervention allocation), a different masked assessor took over.

### Contamination

In order to assess the extent of contamination between groups, at T1, the intervention group was asked about the extent to which they shared information about the intervention, and the control group was asked about whether they heard about the intervention from others.

### Protocol deviation

Minor deviations from, or additions to, the protocol (Brown et al., 2022) were recorded and include: a family accidentally assessed twice at baseline (the most recent assessment was used in the analysis); two families experienced the death of a caregiver during the trial and were provided with additional support from facilitators; two families received the booster session before T1 assessment instead of after; T1 assessments for 6 control group families were done 4 weeks early due to ethical concerns that they would otherwise not receive the program before the end of the project; two families had the same father (both were included in the analysis using mother report only). In terms of external events, assessments were delayed in early 2022 due to a storm affecting transport and phone connections, and assessment compensation to participants had to be changed twice due to major drops in the value of local currency.

### Analysis

All analyses were detailed in a statistical analysis plan, signed before unmasking the study data set. To determine comparability between the conditions at baseline, multiple planned comparisons were conducted, and significance testing was adjusted for multiple comparisons. A primary analysis strategy of an intent‐to‐treat approach was used to test for statistically significant differences over time on primary and secondary outcomes between the two conditions. Hierarchical linear modeling (HLM) was conducted for each continuous outcome, using a group × time interaction analysis. Fixed effects parameters were tested for intervention conditions and time of assessments at 95% confidence intervals. Level 1 of the model represented within‐patient change over time on the outcomes of interest, and Level 2 of the model included variables in which the participants were nested to provide better estimates of the within‐patient change over time. Covariates were added and model fit parameters were used to determine the best‐fitting models for our data. Across all analyses, two‐tailed tests were reported with *p* < .05. Three‐month follow‐up data were tested by comparing within‐group differences between T2 and T3 in the intervention group, adjusted for T1. Per protocol secondary analyses were also conducted, whereby only ‘treatment completers’ (pre‐defined as having completed 4 sessions or more) were included. In addition, we ran a moderation analysis using the ‘lmer’ package. The model specified gender, age, nationality, father attendance, working status, past traumatic exposures, level of adversity, and income. It was based on interaction effects with group allocation, the implementing partner was set as a random effect and change in adolescent well‐being between T0 and T1 as the outcome variable.

## Results

### Participant characteristics

As shown in Table 1, the index adolescents were split evenly between girls and boys (53.78% female), with an average age of 14.19 (*SD* = 1.67), and more than a third were in paid work (38.67%), predominantly in agriculture. The majority of caregivers were Syrian (72.10%), with an average monthly income of less than 850,000 LBP (between 102 USD in November 2020 and 69 USD in April 2021). The adolescent sample had an average of 18.48 (*SD* = 2.51) adverse events and 5.07 (*SD* = 3.38) potentially traumatic events in their lifetimes. The majority of adolescents experienced a lack of sufficient food or water (57.26%), poor/unsafe housing conditions (56.70%), living in a war zone (70.66%), and danger during flight (65.53%) (details can be found in Table S3). There were no statistically significant differences between intervention and control in any of the primary and secondary outcomes for adolescents or caregivers at baseline.

**Table 1:** Participant characteristics at baseline, by intervention allocation

Characteristic^a^		Total (*n* = 351)	Family intervention (*n* = 174)	Waitlist control (*n* = 177)
Age of adolescent (*M*, *SD*)		14.19 (1.67)	14.22 (1.6)	14.16 (1.72)
Age of caregivers (*M*, *SD*)		42.56 (7.35)	42.06 (7.17)	43.06 (7.52)
Gender of adolescent (*M*, %)	Female	188 (53.78)	98 (56.32)	90 (50.85)
Gender of caregivers (*M*, %)	Female	320 (82.47) (*n* = 388 total caregivers)	159 (81.54) (*n* = 195 total caregivers)	161 (83.42) (*n* = 193 total caregivers)
Adolescent completion (4+ sessions)		246 (70.09%)	125 (71.84%)	121 (68.36%)
Fathers attended 1+ sessions *n* (%) for intervention (family sessions) and control (multi‐family sessions)		92 (26.74)	67 (38.95)	25 (14.53)
Nationality of parents (*n*, %)	Lebanese	82 (23.36)	41 (23.56)	41 (23.16)
	Syrian	253 (72.10)	123 (70.69)	130 (73.45)
	Palestinian	3 (0.85)	2 (1.15)	1 (0.56)
	Mixed nationalities	13 (3.70)	8 (4.60)	5 (2.82)
Household income in LBP per month (*M*, *SD*)		843,088.20 (979,925.40)	791,190.50 (783,328.50)	891,069.20 (1,132,180)
Number of adolescents working^b^ (*M*, *SD*)		137 (38.67)	66 (37.93)	71 (40.11)
Types of adolescent work (*n*, %)	Agriculture	53 (38.67)	25 (37.88)	28 (39.44)
	Mechanic	6 (4.38)	3 (4.55)	3 (4.23)
	Selling on the street	30 (21.90)	12 (18.18)	18 (25.35)
	Supermarket	10 (7.30)	6 (9.10)	4 (5.63)
	Other	38 (27.74)	20 (30.30)	18 (25.35)
Adverse events lifetime (*M*, *SD*)		18.48 (2.51)	18.44 (2.49)	18.52 (2.53)
Potentially traumatic events lifetime (*M*, *SD*)		5.07 (3.38)	5.09 (3.31)	5.06 (3.45)

^a^ Note that *t*‐test/chi^2^ tests found no significant differences between groups on any characteristic.

^b^ Note this measure does not include unpaid household labor.

### Effectiveness of the Sawa Aqwa family intervention

Within‐group analyses in the intervention group show significant improvement for all primary and secondary outcomes (shown in Table 2 for adolescents and Table 3 for caregivers). The primary strategy of intent‐to‐treat analyses compared the change between T0 and T1 by allocated condition (presented in Table 4), with mixed effects models (condition set as fixed effects and the implementing partner as random effects). The intervention group improved significantly more than the control group on adolescent‐reported family functioning (*β* = 2.11, *p* = .02), caregiver mental health (*β* = 1.67, *p* = .00), caregiver well‐being (*β* = 1.79, *p* = .00), and parenting (*β* = 1.69, *p* = .01). No between‐group differences were found for other outcomes.

**Table 2:** Within‐group outcomes for adolescents

Outcome and baseline alpha	Control				Intervention			
	Baseline (*n* = 174)	Endline (*n* = 168)	Within group *t*‐test	*d*	Baseline (*n* = 177)	Endline (*n* = 154)	Within group *t*‐test	*d*
	*M* (*SD*)	*M* (*SD*)			*M* (*SD*)	*M* (*SD*)		
PSC (*a* = .71)	33.1 (7.89)	30.2 (8.91)	*t*(167) = −5.03, *p* < .001	.39	31.9 (7.16)	30.1 (9.35)	*t*(152) = −2.38, *p* = .018	.19
WHO‐5 (*a* = .77)	10.3 (6.17)	11.1 (5.86)	*t*(167) = 1.55, *p* = .12	.12	10.6 (6.18)	13 (5.83)	*t*(152) = 4.13, *p* < .001	.33
SCORE (*a* = .80)	42.3 (8.08)	41.2 (9.02)	*t*(162) = −2.07, *p* = .04	.16	42.2 (7.06)	39.6 (7.78)	*t*(151) = −3.97, *p* = .0001	.32
DERS (*a* = .75)	57.5 (11.2)	55.4 (12.2)	*t*(162) = −2.48, *p* = .014	.19	56.5 (11.0)	52.8 (11.8)	*t*(151) = −3.56, *p* = .0005	.29

DERS, Difficulties in Emotion Regulation Scale; K6, Kessler Psychological Distress Scale, PQ, Parenting Questionnaire; PSC, Pediatric Symptom Checklist; SCORE, Systemic Clinical Outcome and Routine Evaluation; WHO, Wellbeing Index.

**Table 3:** Within‐group outcomes for caregivers

Outcome and baseline alpha	Control				Intervention			
	Baseline T0	Endline T1	Within group *t*‐test	*d*	Baseline T0	Endline T1	Within group *t*‐test	*d*
	(*n* = 193)	(*n* = 190)			(*n* = 195)	(*n* = 191)		
	*M* (*SD*)	*M* (*SD*)			*M* (*SD*)	*M* (*SD*)		
K6 (*a* = .90)	14.2 (4.52)	14.7 (4.9)	*t*(183) = 1.87, *p* = .06	.14	14.7 (4.44)	16.9 (4.87)	*t*(172) = 5.22, *p* < .001	.40
WHO‐5 (*a* = .91)	7.89 (5.80)	8.44 (5.58)	*t*(184) = 1.02, *p* = .31	.08	8.79 (5.77)	11.1 (5.88)	*t*(173) = 4.65, *p* < .001	.35
SCORE (*a* = .94)	43.0 (8.87)	42.1 (8.82)	*t*(184) = −2.34, *p* = .02	.17	42.4 (7.81)	40.2 (7.31)	*t*(173) = −4.61, *p* < .001	.35
DERS (*a* = .91)	56.3 (11.5)	54.8 (12.2)	*t*(180) = −2.20, *p* = .03	.16	55 (11.9)	53.3 (10.9)	*t*(172) = −2.35, *p* = .02	.18
Parenting (*a* = .95)	60.7 (7.10)	60.8 (8.20)	*t*(184) = 0.53, *p* = .6	4	61.5 (6.93)	63.3 (5.63)	*t*(173) = 3.92, *p* < .001	.30

**Table 4:** Intent‐to‐treat analysis

Outcome	Control		Intervention		
	Baseline	Endline	Baseline	Endline	
	*M* (*SD*)	*M* (*SD*)	*M* (*SD*)	*M* (*SD*)	
Adolescents					
PSC	33.1 (7.89)	30.8 (9.05)	31.9 (7.16)	31.2 (9.22)	*β* = 1.56, *p* = 08
WHO‐5	10.3 (6.17)	10.9 (5.78)	10.6 (6.18)	12.5 (5.63)	*β* = 1.34, *p* = .07
SCORE	42.3 (8.08)	42.0 (9.34)	42.2 (7.06)	39.7 (7.32)	*β* = −2.11, *p* = .02
DERS	57.5 (11.2)	56 (12.2)	56.5 (11)	53.9 (11.6)	*β* = −.10, *p* = .47
Caregivers					
K6	14.2 (4.52)	14.7 (4.96)	14.7 (4.43)	16.8 (4.74)	*β* = 1.67, *p* = .00
WHO‐5	7.89 (5.80)	8.44 (5.54)	8.79 (5.77)	11.1 (5.85)	*β* = 1.79, *p* = .00
SCORE	43.0 (8.87)	41.8 (8.97)	42.4 (7.81)	40.1 (7.21)	*β* = −1.09, *p* = .15
DERS	56.3 (11.5)	54.3 (12.2)	55 (11.9)	53.1 (10.8)	*β* = −0.01, *p* = .9
Parenting	60.7 (7.10)	60.9 (8.12)	61.5 (6.93)	63.4 (5.56)	*β* = 1.69, *p* = .01

Secondary per protocol analyses showed that adolescent ‘completers’ improved significantly more on well‐being than the control group (*β* = 1.77, *p* = .02). For caregiver ‘completers’, mothers in the intervention group improved significantly more than the control group on mental health (*β* = 1.61, *p* = .01), well‐being (*β* = 1.81, *p* = .02), and parenting (*β* = 1.47, *p* = .05), while fathers in the intervention group improved significantly more than the control group on parenting (*β* = 2.99, *p* = .01). Findings did not change when including only adolescents who reported high distress at baseline.

#### Moderating factors

Father participation (number of sessions attended) moderated the strength of the relationship between treatment condition and adolescent well‐being (*t* = 2.81, *p* = .01). No other moderators were found for adolescents or caregivers, as shown in Table 5. A post hoc power calculation using G*Power indicated the moderation analysis is likely underpowered (73%).

**Table 5:** Moderation analysis

Independent variable	Adolescent well‐being	
	*t* Value	*p*
Condition	−0.70	.48
Gender	0.27	.78
Age	−1.82	.07
Syrian nationality	0.39	.70
Other non‐Lebanese nationality	−0.90	.37
Adolescent working	0.97	.33
Past trauma exposure	0.64	.52
Adversity events	0.54	.59
Income	−0.91	.37
Father attendance (1+ sessions)	−1.922	.06
Interaction group*age	1.51	.13
Interaction group*gender	−0.76	.45
Interaction group*Syrian nationality	−0.16	.88
Interaction group*other nationality	0.01	.99
Interaction group*working	−1.20	.23
Interaction group*trauma	−0.21	.84
Interaction group*adversity	−0.31	.75
Interaction group*income	0.75	.45
Interaction group*father attendance	2.81	.005

#### Three‐month follow‐up

At 3‐month follow‐up, gains on all adolescent and caregiver outcomes were maintained, with no reductions compared to endline scores, except for adolescent‐reported family functioning (*t*(171) = 3.57, *p* < .01) and caregiver well‐being (*t*(153) = −2.61, *p* = .01).

### Masking and contamination

Assessor masking was successfully maintained. Assessors were not able to guess the condition allocation of assessed families (correct guess 54%, exact binomial test *p* = .08). Contamination (sharing or receiving intervention materials) was limited, with only 1 family receiving material while in the control and 42 intervention families sharing materials with others. Fourteen families in the control and 10 in the intervention accessed other kinds of services during the trial.

## Discussion

This study assessed the effectiveness of a family systemic psychosocial support intervention, Sawa Aqwa (Stronger Together), deliverable by non‐specialists to families of adolescents through existing child protection systems in a humanitarian setting. The trial did not find evidence of the effectiveness of the intervention in reducing adolescent psychological distress (the primary outcome). The study did, however, show secondary evidence for improving adolescent well‐being, caregiver mental health and well‐being, family functioning, and parenting. The latter findings are in line with the intervention's Theory of Change that links improving family functioning to improvements in caregiver and adolescent well‐being, as well as to safety through improved parenting practices. However, no changes were seen on adolescent‐ or caregiver‐reported emotional regulation, nor on caregiver‐reported family functioning. At 3‐month follow‐up, we found the maintenance of gains for adolescent‐reported well‐being and caregiver‐reported mental health and parenting, providing promising evidence of durability. However, further testing is required with a control group at follow‐up and a longer follow‐up.

The lack of significant intervention effects on the primary outcome of adolescent psychological distress contrasts with our expectations from our Theory of Change, and findings of similar interventions in other low‐resource settings that have found significant changes for adolescent mental health. These include the Happy Families Parenting and Family Skills Intervention in Thailand (Puffer, Annan, Sim, Salhi, & Betancourt, 2017), Project Parceria in Brazil (Santini & Williams, 2017), and the Sinovuyo Caring Families Teen Programme in South Africa (Cluver et al., 2017). However, most of these interventions were child not adolescent‐focused and not in a humanitarian or war‐affected setting. Additionally, all were preventative, without our focus on adolescents with established psychological distress and child protection vulnerability. Our sample also had high exposure to major life adversities, including war and displacement. In Lebanon, other adolescent mental health‐focused interventions have similarly found no evidence of change on mental health (e.g., Jordans et al., 2023). However, these studies were underpowered and stopped prematurely due to the COVID‐19 pandemic and civil unrest, demonstrating the major challenges of conducting research in Lebanon. Still other interventions that similarly did not find significant changes for externalizing and internalizing difficulties at endline, did find improvements for prosocial behaviors (such as the Family Strengthening Intervention in Rwanda; Betancourt et al., 2014) and risk behaviors (such as the READY family‐based intervention in Kenya; Puffer et al., 2016), which may be more relevant indicators for the age group. Alternatively, there may be a *delayed* effect of improved family functioning and parenting on adolescent mental health. While we found good evidence for an effect on general family well‐being (as indicated by improvements in family functioning, parenting, and adolescent well‐being), we might expect adolescent mental health effects to show later, after a longer exposure to a nurturing family environment. In line with the ‘sleeper effect’ in family systems theory, the effects of interventions can appear or increase over time after intervention. This is thought to occur due to repeated exposure to positive changes in the environment, such as improved parenting, nurturing relationships, and family functioning (van Aar, Leijten, Orobio de Castro, & Overbeek, 2017). This explanation is supported by studies with a longer follow‐up. For example, the Family Strengthening Intervention found no improvement in anxiety and depression at post‐intervention, but they did at 6‐month follow‐up (Betancourt et al., 2014). Family functioning may also play a more of a buffering effect on mental health in the context of Lebanon. Research from the previous 2006 war found that adolescent mental health was buffered by nurturing caregiver relationships (Fayyad et al., 2017) and social support (Yamout & Chaaya, 2011). A longer follow‐up, particularly during times of major adversity, could help to capture any longer‐term benefits to mental health following family functioning improvements.

While implementation findings are presented in full in a separate paper (Bosqui et al., in prep), the experience of delivering the Sawa Aqwa (Stronger Together) Family Intervention—with an exceptionally high uptake and good attendance—provides evidence of the high acceptance and perceived utility of whole‐family approaches and feasibility to implement them within existing child protection services and by non‐specialist providers in Lebanon. The innovative use of ‘task‐sharing approaches’—the training and supervision of non‐specialist providers—has helped to increase the reach and scale of MHPSS interventions in low‐resource settings (Brown, de Graaff, Annan, & Betancourt, 2017). In Lebanon, estimates indicate there are only 1.26 psychiatrists and 3.42 psychologists per 100,000 population, with 97% working in the private sector, making mental health care inaccessible to the most vulnerable (El‐Khoury, Haidar, & Charara, 2020). Maximizing the use of existing non‐specialist providers in the workforce has the potential to reduce this gap.

Finally, we found that higher father attendance in the intervention was associated with greater adolescent well‐being, albeit with a small effect size. This provides preliminary evidence of the importance of engaging fathers in interventions for adolescents. Assumed gendered roles have long led to an over‐focus on mothers as the primary or only influence on adolescents, despite consistent and convincing evidence to the contrary (Mestermann et al., 2023). Paternal involvement in the family has been linked to better maternal well‐being and lower parenting stress and harsh discipline (Hein et al., 2020), lower intimate‐partner violence (Betancourt, Jensen, & Barnhart, 2020), and lower levels of adolescent mental health difficulties (Culpin et al., 2022). A review of family interventions identified the engagement and attendance of fathers as critical to the success of intervention access and delivery (Bosqui et al., 2024). This suggests that the active engagement of fathers in interventions focused on adolescent health and well‐being may be crucial, and that further efforts to remove barriers to father participation deserve attention.

### Strengths and limitations

This study benefits from a contextually‐grounded intervention developed in collaboration with the target population, evaluated through a multi‐site RCT design, and with a sufficient sample size. The hybrid design allowed for the inclusion of both effectiveness and implementation data. Limitations include a relatively short follow‐up of 3 months, preventing an analysis of longer‐term maintenance of gains and any delayed improvements, such as for adolescent psychological distress. Moderation analyses may also have been underpowered as moderation was not included in the initial sample size calculation. In addition, greater focus on implementation science in future work could support efforts to increase reach and scale, and an equivalence design to compare the intervention to active controls of other evidence‐based MHPSS interventions for adolescents. Findings may be generalizable for use with the same target population in Lebanon, but further research is needed in the region to generalize findings further (such as in Jordan, Brown et al., 2024).

## Conclusions

Despite clear evidence for the importance of the family system for adolescent mental health, well‐being, and healthy development into adulthood, few whole‐family interventions have been developed and tested in the context of ongoing humanitarian crises. The Sawa Aqwa Family Intervention aimed to address this gap. While we did not find evidence of effectiveness for adolescent psychological distress, we did find evidence for effectiveness on other outcomes related to family functioning, parenting, and well‐being. This evidence should be used to inform humanitarian programming in mental health and psychosocial support to better meet the systemic family needs of adolescents affected by humanitarian emergencies and displacement.

## Ethical considerations

Informed assent from children and informed consent from parents or legal guardians have been appropriately obtained. The trial received ethical approval from the Human Research Protection Program's Institutional Review Board of the American University of Beirut (Reference: SBS‐2021‐0102, approved on 12 October 2021), and from the National Mental Health Program in the Ministry of Public Health in Lebanon (Approved on the 19th October 2021).

## Trial registration

The trial was registered with the Lebanese Clinical Trial Registry (registered 13th October 2021, reference: LBCTR2021104870, available from https://lbctr.moph.gov.lb/LBCTR/Trials/Details/4979) and the ISRCTN (registered 1st November 2021, reference: ISRCTN13751677, available from https://doi.org/10.1186/ISRCTN13751677).

**Key points**

**What's known?**

- Nurturing family environments are essential for adolescent mental health but are rarely addressed in mental health and psychosocial support (MHPSS) interventions in humanitarian settings.

**What's new?**

- The Sawa Aqwa Family Intervention is effective for improving family functioning, caregiver and adolescent well‐being, and parenting.

**What's relevant?**

- A whole‐family approach is an effective way to address the wider social ecology of adolescents and can be integrated into existing child protection systems.

## Supporting information


**Table S1.** CONSORT 2010 checklist of information to include when reporting a randomized trial.
**Table S2.** Summary of the Sawa Aqwa (Stronger Together) family intervention.
**Table S3.** Index Adolescent Adversity at baseline, by intervention allocation.

## Data Availability

The data that support the findings of this study, including deidentified individual clinical trial participant‐level data, are available on request from T.B., American University of Beirut, and M.J., War Child Alliance. The data are not publicly available due to privacy or ethical restrictions.
