# Supplementary material for: ‘Sawa Aqwa’ (Stronger Together): A multi‐site randomized controlled trial of a brief family systemic intervention for adolescent mental health in Lebanon
Source: J Child Psychol Psychiatry. 2026 Jun 4;67(10):1752–65. doi: 10.1111/jcpp.70174 (PMC13535759; doi:10.1111/jcpp.70174)
Supplement: Supplementary file 1 [file JCPP-67-1752-s001.docx]

**“Sawa Aqwa” (Stronger Together): A multi-site randomised controlled trial of a**

**brief family systemic intervention for adolescent mental health in Lebanon**

**Supporting Information**

Table S1. *CONSORT 2010 checklist of information to include when reporting a randomised trial**

| Section/Topic | Item No | Checklist item | Reported on page No |
| --- | --- | --- | --- |
| Title and abstract | | | |
|  | 1a | Identification as a randomised trial in the title | 1 |
|  | 1b | Structured summary of trial design, methods, results, and conclusions (for specific guidance see CONSORT for abstracts) | 2 |
| Introduction | | | |
| Background and objectives | 2a | Scientific background and explanation of rationale | 3-4 |
|  | 2b | Specific objectives or hypotheses | 5 |
| Methods | | | |
| Trial design | 3a | Description of trial design (such as parallel, factorial) including allocation ratio | 5 |
|  | 3b | Important changes to methods after trial commencement (such as eligibility criteria), with reasons | N/A |
| Participants | 4a | Eligibility criteria for participants | 7 |
|  | 4b | Settings and locations where the data were collected | 8 |
| Interventions | 5 | The interventions for each group with sufficient details to allow replication, including how and when they were actually administered | 9 |
| Outcomes | 6a | Completely defined pre-specified primary and secondary outcome measures, including how and when they were assessed | 10-11 |
|  | 6b | Any changes to trial outcomes after the trial commenced, with reasons | N/A |
| Sample size | 7a | How sample size was determined | 8 |
|  | 7b | When applicable, explanation of any interim analyses and stopping guidelines | N/A |
| Randomisation: |  |  |  |
| Sequence generation | 8a | Method used to generate the random allocation sequence | 9 |
|  | 8b | Type of randomisation; details of any restriction (such as blocking and block size) | 9 |
| Allocation concealment mechanism | 9 | Mechanism used to implement the random allocation sequence (such as sequentially numbered containers), describing any steps taken to conceal the sequence until interventions were assigned | 13-14 |
| Implementation | 10 | Who generated the random allocation sequence, who enrolled participants, and who assigned participants to interventions | 9, 13-14 |
| Blinding | 11a | If done, who was blinded after assignment to interventions (for example, participants, care providers, those assessing outcomes) and how | N/A |
|  | 11b | If relevant, description of the similarity of interventions | N/A |
| Statistical methods | 12a | Statistical methods used to compare groups for primary and secondary outcomes | 15 |
|  | 12b | Methods for additional analyses, such as subgroup analyses and adjusted analyses | 15 |
| Results | | | |
| Participant flow (a diagram is strongly recommended) | 13a | For each group, the numbers of participants who were randomly assigned, received intended treatment, and were analysed for the primary outcome | 15-16 |
|  | 13b | For each group, losses and exclusions after randomisation, together with reasons | 15-16, Fig 1 |
| Recruitment | 14a | Dates defining the periods of recruitment and follow-up | 15 |
|  | 14b | Why the trial ended or was stopped | N/A |
| Baseline data | 15 | A table showing baseline demographic and clinical characteristics for each group | Table 1 |
| Numbers analysed | 16 | For each group, number of participants (denominator) included in each analysis and whether the analysis was by original assigned groups | 16 |
| Outcomes and estimation | 17a | For each primary and secondary outcome, results for each group, and the estimated effect size and its precision (such as 95% confidence interval) | 16-17 |
|  | 17b | For binary outcomes, presentation of both absolute and relative effect sizes is recommended | N/A |
| Ancillary analyses | 18 | Results of any other analyses performed, including subgroup analyses and adjusted analyses, distinguishing pre-specified from exploratory | 17 |
| Harms | 19 | All important harms or unintended effects in each group (for specific guidance see CONSORT for harms) | 17 |
| Discussion | | | |
| Limitations | 20 | Trial limitations, addressing sources of potential bias, imprecision, and, if relevant, multiplicity of analyses | 20 |
| Generalisability | 21 | Generalisability (external validity, applicability) of the trial findings | 20 |
| Interpretation | 22 | Interpretation consistent with results, balancing benefits and harms, and considering other relevant evidence | 18-19 |
| Other information | | |  |
| Registration | 23 | Registration number and name of trial registry | 5 |
| Protocol | 24 | Where the full trial protocol can be accessed, if available | 5 |
| Funding | 25 | Sources of funding and other support (such as supply of drugs), role of funders | 21 |

Citation: Schulz KF, Altman DG, Moher D, for the CONSORT Group. CONSORT 2010 Statement: updated guidelines for reporting parallel group randomised trials. BMC Medicine. 2010;8:18.
© 2010 Schulz et al. This is an Open Access article distributed under the terms of the Creative Commons Attribution License (<http://creativecommons.org/licenses/by/2.0>), which permits unrestricted use, distribution, and reproduction in any medium, provided the original work is properly cited.

*We strongly recommend reading this statement in conjunction with the CONSORT 2010 Explanation and Elaboration for important clarifications on all the items. If relevant, we also recommend reading CONSORT extensions for cluster randomised trials, non-inferiority and equivalence trials, non-pharmacological treatments, herbal interventions, and pragmatic trials. Additional extensions are forthcoming: for those and for up-to-date references relevant to this checklist, see [www.consort-statement.org](http://www.consort-statement.org).

**Table S2**

*Summary of the Sawa Aqwa (Stronger Together) Family Intervention*

| **Session** | **Aims** | **Who** | **Content** |
| --- | --- | --- | --- |
| **1**  **Our Family Story** | 1. Family develops joint understanding of challenges, goals, and strengths of family 2. Family develops joint understanding of roles and responsibilities 3. Caregivers are encouraged to work together as a team | Family | - Introduction to programme- 15 minutes - Confidentiality- 10 minutes - Ground rules- 10 minutes - Family timeline- 40 minutes - Reflection and sharing and setting home practice- 10 minutes - Closing- appreciation statements- 5 minutes |
|  |  | Caregivers | - Gather more information on parenting challenges - Working together as a parenting team |
| **2**  **How Our Family Wants to Be** | 1. Family develops joint understanding of challenges, goals, and strengths of family 2. Family understands each other and builds empathy | Family | - Opening activity- 10 minutes - Review and feedback- 15 minutes - Introducing metaphor and identifying family values- 50 minutes - Reflection and sharing and setting home practice– 10 minutes - Closing- appreciation statements- 5 minutes |
|  |  | Caregivers | - Identifying parenting strengths, challenges, and values - Setting goal around spending quality time with adolescents |
| **3**  **Managing Difficult Feelings and Helping Each Other** | 1. Family members build awareness of feelings experienced by themselves and others 2. Family builds skills in grounding, and stepping back from thoughts, to help them manage difficult thoughts and feelings 3. Family learns how to practice good self-care for positive wellbeing | Family | - Opening activity- 10 minutes - Review and feedback- 15 minutes - Identifying and managing feelings- 50 minutes - Reflection and sharing and setting home practice– 10 minutes - Closing- appreciation statements- 5 minutes |
|  |  | Caregivers | - Noticing emotions in children - Supporting children with big and difficult feelings |
| **4.  Communicating Well to Support Each Other** | 1. Family understands their common barriers to positive communication 2. Family builds skills in positive communication, including good listening, and expressing themselves | Family | - Opening activity- 10 minutes - Review and feedback- 15 minutes - Listening and Expressing Ourselves- 50 minutes - Reflection and sharing and setting home practice– 10 minutes - Closing- appreciation statements- 5 minutes |
|  |  | Caregivers | - Praising children for their efforts - Showing appreciation for children and partners |
| **5.**  **Managing Our Problems Together** | 1. Family identifies common problems they are facing 2. Family learns and applies Stop Think Go problem management steps to their problems | Family | - Opening activity- 10 minutes - Review and feedback- 15 minutes - Managing our problems together- 50 minutes - Reflection and sharing and setting home practice– 10 minutes - Closing- appreciation statements- 5 minutes |
|  |  | Caregivers | - Managing problems as caregivers - Caregivers as teachers - Giving independence and setting limits with adolescents |
| **6.**  **Managing Problems When We Don’t All Agree** | 1. Family discusses strengths, roles and responsibilities in the family 2. Family identifies common sources of conflict 3. Family learns and applies Stop Think Go problem solving steps to problems involving disagreements between family members | Family | - Opening activity- 10 minutes - Review and feedback- 15 minutes - Family strengths and family roles- 20 minutes - Managing problems involving disagreements- 30 minutes - Reflection and sharing and setting home practice– 10 minutes - Closing- appreciation statements- 5 minutes |
|  |  | Caregivers | - Psychoeducation on changes during adolescence - Working as a team with adolescents and managing disagreements |
| **Booster:**  **Reviewing Where we Started and Where we Want to go in the Future!** | 1. Family reviews content learned in sessions 2. Family reviews home practice of strategies and trouble shoots any challenges they have faced 3. Family makes a plan for the future, maintaining progress made through these sessions | Family | - Opening activity- 10 minutes - Strategy review- 20 minutes - Looking to the future- 15 minutes - Social support- 15 minutes - Graduation ceremony- 30 minutes |

**Table S3**

*Index Adolescent Adversity at Baseline, by Intervention Allocation*

| **Event type** |  | **Total (*n*=351)** | **Family intervention (*n*=174)** | **Waitlist control (*n*=177)** |
| --- | --- | --- | --- | --- |
| **Adverse events lifetime *(n,* %)** |  |  |  |  |
|  | Inadequate food or water | 201 (57.26%) | 100 (57.47) | 101 (57.06) |
|  | Poor/unsafe housing conditions | 199 (56.70) | 98 (56.32) | 101 (57.06) |
|  | Forced to leave home | 112 (31.91) | 55 (31.61) | 57 (32.20) |
|  | Lack of any shelter | 100 (28.49) | 47 (27.01) | 53 (29.94) |
|  | Chronic illness | 44 (12.54) | 24 (13.79) | 20 (11.30) |
|  | Serious chronic illness of family member | 83 (23.65) | 39 (22.41) | 44 (24.86) |
|  | Ill health without access to medical care | 124 (35.33) | 60 (34.48) | 64 (36.16) |
|  | Difficult working conditions | 113 (32.19) | 52 (29.89) | 61 (34.46) |
|  | Use of dangerous machinery in  workplace | 37 (10.54) | 17 (9.77) | 20 (11.30) |
|  | Sexual harassment | 63 (17.95) | 31 (17.82) | 32 (18.08) |
|  | Physical violence by a family member | 49 (13.96) | 26 (14.94) | 23 (12.99) |
|  | Witnessing physical violence between family members | 58 (16.52) | 24 (13.79) | 34 (19.21) |
|  | Being shouted at, insulted or bullied by a family member | 74 (21.08) | 33 (18.97) | 41 (23.16) |
|  | Being shouted at, insulted or bullied by any other person | 108 (30.77) | 52 (29.89) | 56 (31.64) |
|  | Parental divorce or separation | 35 (9.97) | 13 (7.47) | 22 (12.43) |
|  | Social isolation | 123 (35.04) | 57 (32.76) | 66 (37.29) |
| **Potentially traumatic events lifetime *(n, %)*** |  |  |  |  |
|  | Lived in a refugee camp | 168 (47.86) | 81 (46.55) | 87 (49.15) |
|  | Lived in a war zone | 248 (70.66) | 124 (71.26) | 124 (70.06) |
|  | Danger during flight | 230 (65.53) | 116 (66.67) | 114 (64.41) |
|  | Serious physical injury | 38 (10.83) | 15 (8.62) | 23 (12.99) |
|  | Serious physical injury of family member | 72 (20.51) | 36 (20.69) | 36 (20.34) |
|  | Life-threatening illness | 16 (4.56) | 9 (5.17) | 7 (3.95) |
|  | Exposure to toxic substance | 28 (7.98) | 12 (6.90) | 16 (9.01) |
|  | Serious accidents or explosion | 88 (25.07) | 49 (28.16) | 39 (22.03) |
|  | Forced separation from family member | 54 (15.38) | 26 (14.94) | 28 (15.82) |
|  | Kidnapped/attempted kidnap | 31 (8.83) | 14 (8.05) | 17 (9.6) |
|  | Forced to hide | 176 (50.14) | 89 (51.15) | 87 (49.15) |
|  | Sexual assault | 21 (5.98) | 11 (6.32) | 10 (5.65) |
|  | Witnessed  rape or sexual abuse | 7 (1.99) | 2 (1.15) | 5 (2.82) |
|  | Physical assault by any other person | 79 (22.51) | 42 (24.14) | 37 (20.90) |
|  | Witnessed physical assault towards another person | 136 (38.75) | 66 (37.93) | 70 (39.55) |
|  | Witnessed killing/murder of another person | 65 (18.52) | 33 (18.97) | 32 (18.10) |
|  | Disappearance or kidnapping of family member or friend | 50 (14.25) | 21 (12.07) | 29 (16.38) |
|  | Family member or friend murdered or died due to violence or suicide | 50 (14.25) | 22 (12.64) | 28 (15.82) |
|  | Seen a dead body | 121 (34.47) | 60 (34.48) | 61 (34.46) |
|  | Other situation that was frightening or felt life was in danger | 101 (28.77) | 52 (29.89) | 49 (27.68) |
